# Rethinking One Health approach in the challenging era of COVID-19 pandemic and natural disasters

**DOI:** 10.1080/20008686.2020.1852681

**Published:** 2020-11-23

**Authors:** Hajime Kanamori, Hiroaki Baba, David J. Weber

Dear Editor:

Emergency response in a natural disaster causing evacuation, transportation, and mass gathering in the affected areas may contrast prevention strategies in the COVID-19 pandemic, including physical distancing and home isolation[1]. The Sendai Framework for Disaster Risk Reduction 2015–2030, which was adopted at the Third UN World Conference, proposed protection of livelihoods and productive assets (e.g., livestock)[2]; thus, developing disaster preparedness plans/responses using the One Health multidisciplinary approach is urgently needed as part of pandemic planning. In the challenging era of COVID-19 and natural disasters, the One Health approach on human–animal–environmental interface to infectious diseases, antimicrobial resistance, and noncommunicable diseases are necessary. Amuasi et al. proposed the three dimensional approach of shared environment, safe food systems, and shared medicines/interventions at each interface [3]. We have incorporated the three-dimensional concept into the One Health approach in the COVID-19 pandemic (Table 1).

**Table 1.** Health approach of shared environment, safe food systems, and shared medicines/interventions in the COVID-19 pandemic

Animal–environment–human interface	Examples of One Health approach to COVID-19
Shared environment	Identifying intermediate host(s) of SARS-CoV-2, cross-species transmission, and zoonotic transmission. Assessing human-to-animal transmission of SARS-CoV-2 to companion animals (e.g. dogs and cats), animals raised for commercial purposes (e.g. mink, cattle, pigs), wild/zoo animals (e.g. tigers), and impact of infection on these animals. Assessing whether companion animal-to-human transmission of SARS-CoV-2 is possible. Evaluating the persistence of SARS-CoV-2 on environmental surfaces, wastewater, and the air, and modeling the association of SARS-CoV-2 in the ecosystem.
Safe food and food system	Clarifying the impact of the COVID-19 pandemic on current food systems. Establishing alternative food systems in terms of bioactive ingredients, food safety and security, and sustainability.
Shared medicines/interventions	Investigating medicines for treating COVID-19 originated from animal or plant agriculture. Using animal models for the development of antiviral therapy and vaccines for SARS-CoV-2. Developing the surveillance system for SARS-CoV-2 on humans, animals, and the environment.

As seen in the early cases of COVID-19 potentially associated with the seafood market where seafood, bats, marmots, and snakes were available, the food system has challenges for bioactive ingredients, food safety and security, and sustainability[4]. The transmission route of COVID-19 is mainly human-to-human, but some animals (e.g., cats, dogs, tigers, and minks) that had close contact with humans became infected with SARS-CoV-2, that in some cases led to a mild to severe respiratory disease[5]. Although SARS-CoV-2 has been identified from the environment, action plans for the environmental aspect of One Health are not well established. Given the prolonged incubation time, virus shedding from asymptomatic and pre-symptomatic cases of COVID-19, and the underestimated incidence and prevalence in resource-limited countries, wastewater-based surveillance may be a promising tool as SARS-CoV-2 RNA is excreted in the feces[6].

The Asia-Pacific region is vulnerable to natural disasters partially due to climate change, seismic activities, and human development[7]. After the 2011 Great East Japan Earthquake, many survivors were forced to live in evacuation centers under unsanitary conditions leading to infectious disease outbreaks[8]. Staying at a shelter during the COVID-19 pandemic likely would lead to an outbreak because of a lack of physical distancing and the inability to wears masks at all times (e.g. eating). The 2011 Fukushima nuclear power plant accident and the 2015 Nepal earthquake affected the agriculture sector and animal welfare, and zoonoses occurred [9,10], necessitating a cooperative approach between human and animal health professionals. As the threats to human health posed by emerging infectious diseases and natural disasters have increased substantially due to global travel, population growth, urbanization, and climate changes, it is essential to rethink the One Health approach based on multisectoral and multidisciplinary aspects.
